# Loss of Voice

**Published:** 1858-03

**Authors:** 


### LOSS OF VOICE.

It is stated that "the younger Spurgeon, a minister of great promise, has lost his voice, and is sinking into consumption." As he is superior in some respects to his renowned brother, his early death will be regretted by multitudes. Many young clergymen of great promise have prematurely died, in consequence of their own mismanagement, in one or two particulars.

They speak on too high a key from the beginning, not giving the lungs time to warm up, to bring themselves up to the effort. A good traveller does not start on a trot, when he wants his horse to make a good journey that day. John Newland Maffit could speak with apparently a slight effort at the end of an hour's sermon, loud enough to be heard by thousands; but he always commenced on a low key. His first hymn and prayer were scarcely audible.

The next important point is to cool off very gradually before leaving the assembly, when the discourse is ended—very gradually indeed—and then, even in Summer-time, bundle up well before leaving the house, and walk away quickly. Many an excellent minister has sacrificed life by the neglect of these two precautions.

